# Inflammatory marker changes in a 24-month dietary and physical activity randomised intervention trial in postmenopausal women

**DOI:** 10.1038/s41598-020-78796-z

**Published:** 2020-12-14

**Authors:** G. Masala, B. Bendinelli, C. Della Bella, M. Assedi, S. Tapinassi, I. Ermini, D. Occhini, M. Castaldo, C. Saieva, S. Caini, M. M. D’Elios, D. Palli

**Affiliations:** 1 Cancer Risk Factors and Lifestyle Epidemiology Unit, Institute for Cancer Research, Prevention and Clinical Network (ISPRO), Via Cosimo il Vecchio 2, Florence, Italy; 2 Department of Experimental and Clinical Medicine, University of Florence, Florence, Italy

**Keywords:** Cancer prevention, Disease prevention, Health care

## Abstract

Chronic low-grade inflammation plays a role in the pathogenesis of several chronic diseases including cancer. Physical activity (PA) and diet have been supposed to modulate inflammatory markers. We evaluated the effects of a 24-month dietary and/or PA intervention on plasma levels of pro-inflammatory cytokines, a secondary analysis in the DAMA factorial trial. The 234 study participants (healthy postmenopausal women with high breast density, 50–69 years, non smokers, no hormone therapy) were randomised to four arms: (1) isocaloric dietary intervention mainly based on plant-foods; (2) moderate-intensity PA intervention with at least 1 h/week of supervised strenuous activity; (3) both interventions; (4) general recommendations on healthy dietary and PA patterns. Interleukins (IL)-1α, -1β, -6, tumor necrosis factor-α and C-reactive protein were measured at baseline and at the end of the intervention. Intention-to-treat-analyses were carried out using Tobit regression. Although all cytokines tended to increase over time, after 24 months women in the PA intervention (arms 2 + 3) showed lower levels of IL-1α (exp(β) = 0.66; p = 0.04) and IL-6 (exp(β) = 0.70; p = 0.01) in comparison with women in the control group (arms 1 + 4). No effects of the dietary intervention emerged. In healthy postmenopausal women with high breast density a moderate-intensity PA appears to slow the age-related increase of pro-inflammatory cytokines.

## Introduction

It is widely accepted that chronic low-grade inflammation may play a role in a wide range of age related diseases including cardiovascular diseases, type 2 diabetes and cancer^1,2^.

A higher level of inflammation, associated to the decline in circulating oestrogens, has been documented among postmenopausal women^3–5^. Also obesity and abdominal adiposity have been associated with a pro-inflammatory profile^6^.

Cytokines measured as circulating markers of chronic low-grade inflammation include: interleukin 1 (IL-1), interleukin 6 (IL-6), tumor necrosis factor alpha (TNF-α), and C-reactive protein (CRP). IL-6, a multifunctional cytokine involved in the regulation of immune responses, acute-phase responses, ematopoiesis, and inflammation, is one of the most studied cytokines in relation to aging and the development of chronic diseases^7,8^. TNF-α plays crucial roles in the initiation of the inflammatory cascade^9^. CRP is a sensitive systemic biomarker of inflammation and tissue damage mainly produced by hepatocytes, under transcriptional control by IL-6^10,11^. IL-1 is made up of two major proteins, IL-1α and IL-1β with similar biological properties of coordination of the innate immune and inflammatory responses^12^*.* Several studies support the association of a diet rich in vegetables, fruit, nuts, whole grains and unsaturated fatty acids, and of a Mediterranean Diet pattern, with lower levels of circulating pro-inflammatory cytokines^13–17^. An anti-inflammatory effect of physical exercise and the association between sedentary habits and chronic low-grade inflammation have been shown^18–20^. A modulation of circulating markers of inflammation through modification of dietary^21–23^ and physical activity (PA) habits^21,24–26^, partially mediated by weight reduction, has been reported.

The DAMA (Diet, physical Activity and MAmmography) study is a factorial randomised trial aimed at investigating whether a dietary and/or a PA intervention were able to reduce mammographic breast density (BD) among postmenopausal women with high BD^27^ and therefore at higher breast cancer risk^28^*.* The results of the DAMA trial on BD, the primary outcome of the trial, have been reported recently^29^. Here we report the effects of the dietary and PA interventions on plasma levels of five pro-inflammatory cytokines (IL-1α, IL-1β, IL-6, TNF-α, and CRP), as a secondary outcome of the trial.

## Materials and methods

The DAMA study (trial registration number: ISRCTN28492718, date of trial registration 17/05/2012) is a single-centre randomised intervention trial with a 2 × 2 factorial design*.* The trial was approved by the Ethics Committee of the Local Health Authority “Azienda Sanitaria Firenze”, the research was performed in accordance with relevant guidelines/regulations and an informed consent was obtained from all participants.

The study protocol of the DAMA trial, including the objectives of the interventions and the activities performed, was reported in detail in a previous paper^27^.

### Selection of study participants

Participants were recruited between 2009 and 2010 among postmenopausal women, aged 50–69 years, with a negative mammogram performed in the local screening programme showing a BD > 50% according to the Breast Imaging Reporting and Data System (BI-RADS) classification^30^, no recent (past 12 months) hormone therapy use, never smokers (or former smokers for more than 6 months), without a previous diagnosis of cancer, diabetes, major cardiovascular and neurological diseases or other diseases able to hamper their active participation in the study.

### Baseline and follow-up visits

We invited participants for a visit at baseline and 24 (± 3) months after their enrolment into the study. In both visits trained personnel measured weight, height, hip and waist circumference and collected information on dietary and lifestyle habits through validated questionnaires previously used in the frame of the European Prospective Investigation into Cancer and Nutrition (EPIC) study: the Italian Food Frequency Questionnaire^31^, and the EPIC Lifestyle Questionnaire including a section on PA at work and during leisure time^32^. A fasting venous blood sample was taken at baseline and at follow-up visit. Within 24 h after collection, blood samples were processed into plasma, red cells and buffy-coat aliquots and stored in the liquid nitrogen biological bank of the project.

#### Random assignment

After the baseline visit we randomised each woman to one of the four arms of the study through permuted-block randomisation stratified by age (50–59/60–69 years) and Body Mass Index (BMI) category (< 25/ ≥ 25 kg/m^2^), with a constant block size (n = 4).

#### Dietary intervention (arm #1)

The aim of the dietary intervention was to change the composition of diet, in an isocaloric context (without specific advice on the quantity of food to be consumed), adopting a diet mainly based on plant food, with a low glycemic load, low in saturated- and *trans*-fats and alcohol and rich in antioxidants. The intervention objectives were: (a) substitution of refined grains with whole-grains; (b) at least two portions of vegetables at each meal; (c) consumption of fish at least two–three times/week; (d) fresh and processed red meat less than one time/week; (e) at least three-four portions/week of legumes and pulses; (f) two–three portions/day of fruit; (g) a maximum of one portion/week of cakes or desserts; (h) no more than one portion/day of milk or yogurt and two portions/week of cheese; (i) extra-virgin olive oil as the only dressing and cooking fat; (j) no more than one glass of wine per day.

Participants were invited to attend six group meetings and eight cooking classes during the two years of the study.

#### Physical activity intervention (arm #2)

The aim of PA intervention was to increase moderate daily recreational activities, corresponding to about three MET-hours/day (MET = Metabolic Equivalent), up to one hour/day. Suggested daily activities were walking at moderate pace, biking, etc. Women were also requested to perform at least one hour/week of more strenuous activity (6–10 MET)*.* To achieve this latter objective women were requested to attend weekly one-hour sessions led by qualified personnel in an appropriate fitness facility (97 sessions over the 24-month period). Equipment and schemes for home exercises were also provided. Women were requested to attend six group meetings and six collective walks supervised by the study team.

#### Dietary + Physical activity intervention (arm #3)

Participants were asked to combine arms #1 and #2 activities. To reduce possible contamination, women in this arm had separate dietary and PA sessions from the women randomised to arms #1 or #2, respectively.

#### Control arm (arm #4)

Participants received general advice and printed material on healthy dietary and PA patterns according to updated recommendations on cancer prevention^33^ during a group meeting at the beginning of the study.

### Monitoring the adherence to the intervention

Study participants assigned to the intervention arms were requested to keep 5 written 1-week diaries on diet (arm #1), PA (arm #2), or both (arm #3) during the 24-month intervention period. Dietary diaries encompassed all foods and beverages consumed in a 7-day period. In the PA diaries, women were asked to describe their daily non-occupational activities, to report the number of flights of stairs climbed and to record their steps through a digital pedometer provided at the start of the study. The study personnel reviewed the diaries with the aim to monitor the achievement of the study objectives and to counsel the participants who reported specific difficulties.

### Study subjects characteristics

**Figure 1:**
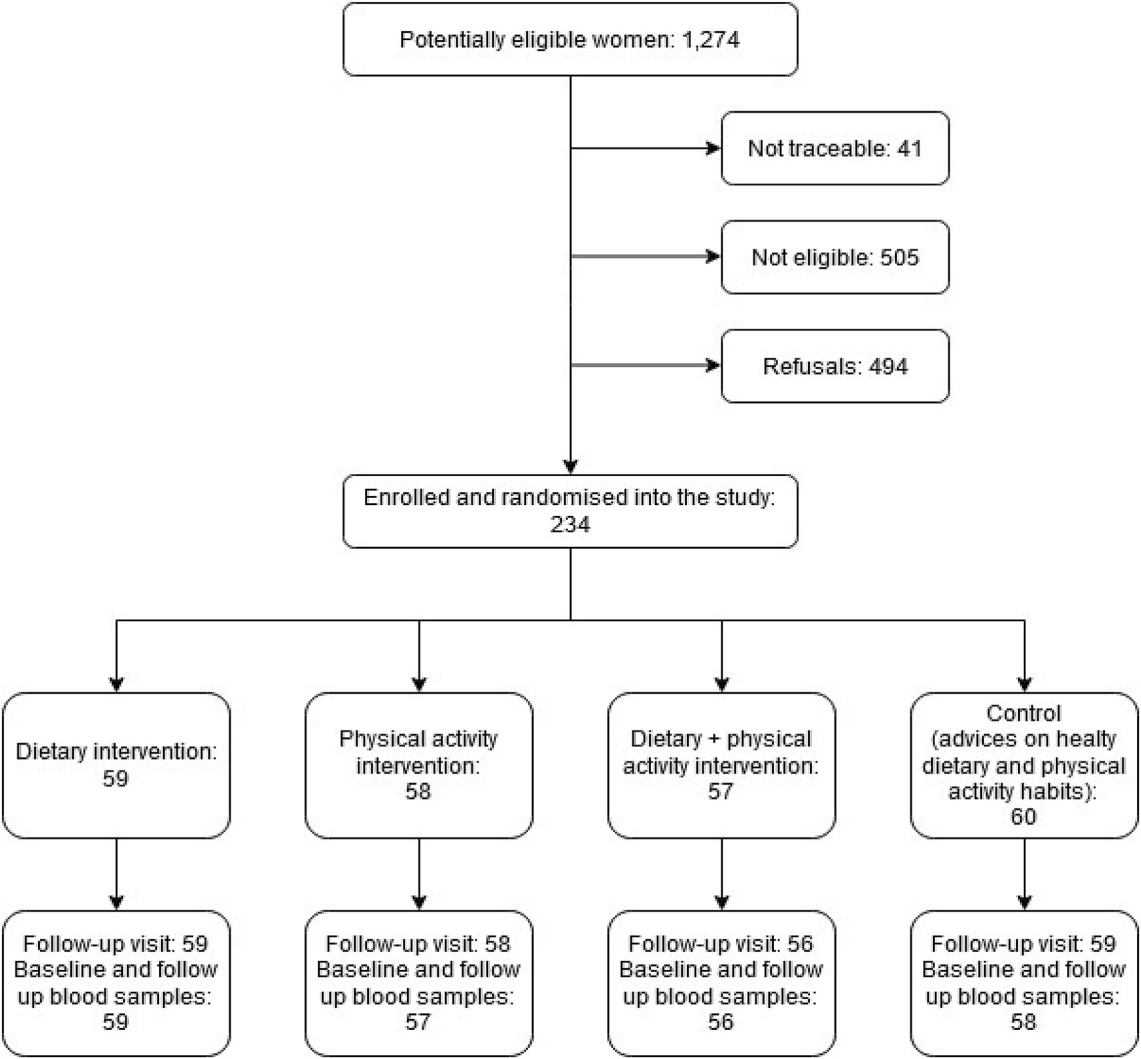
Recruitment of participants, randomisation and follow-up in the DAMA factorial intervention trial. CONSORT Flow Diagram showing procedures of selection among eligible women and participants with complete data available.

We recruited and randomised 234 women of 728 eligible (32.1%). Analyses included 230 women with information and blood samples collected at baseline and follow-up available (Fig. 1). The mean interval between the two visits was 2.0 years (SD 0.1).

The main characteristics of the study participants at baseline were compared by treatment. The mean age of participants was 58.7 year (SD 5.2), with about 70% reporting a high school degree. Most participants were never smokers and normal weight (BMI < 25). Among the 132 (57.4%) women with a paid work, 60.6% reported a sedentary work. The treatment and control groups were similar at baseline for these and other listed variables (Table 1).

**Table 1:** Baseline distribution (categorical variables) or mean and standard deviation (continuous variables) of selected characteristics of women enrolled into the DAMA (Diet, Exercise and Mammography) trial, overall and by treatment.

	Whole study sample (n = 230)	Dietary intervention^a^			Physical activity intervention^b^		
		Treatment Group (N = 115)	Control Group (N = 115)	P value^c^	Treatment Group (N = 113)	Control Group (N = 117)	P value^c^
**Level of education**							
None/primary school	66 (28.7%)	31 (27.0%)	35 (30.4%)		28 (24.8%)	38 (32.5%)	
High school	98 (42.6%)	51 (44.4%)	47 (40.9%)		50 (44.3%)	48 (41.0%)	
University	66 (28.7%)	33 (28.7%)	33 (28.7%)	0.82	35 (31.0%)	31 (26.5%)	0.42
**Smoking**							
Former	97 (42.2%)	44 (38.3%)	53 (46.1%)		46 (39.3%)	51 (45.1%)	
Never	133 (57.8%)	71 (61.7%)	62 (53.9%)	0.27	71 (60.7%)	62(54.9%)	0.38
**General characteristics**							
Age (years)	58.7 (5.2)	58.7 (5.3)	58.7 (5.1)	0.97	58.7 (4.9)	58.6 (5.5)	0.94
Height (cm)	158.9 (6.0)	159.1 (5.8)	158.7(6.3)	0.58	159.2 (5.3)	158.6 (6.7)	0.45
Weight (kg)	61.7 (9.4)	62.3 (9.4)	61.2 (9.4)	0.36	62.6 (10.1)	60.9 (8.7)	0.16
Body Mass Index (kg/m^2^)	24.4 (3.4)	24.6 (3.2)	24.3 (3.7)	0.58	24.7 (3.6)	24.2 (3.3)	0.32
Body Mass Index (groups)							
< 25	145(63.0%)	73 (63.5%)	72 (62.6%)		73 (64.6%)	72 (61.5%)	
≥ 25	85 (37.0%)	42 (36.5%)	43 (37.4%)	0.89	40 (35.4%)	45 (38.5%)	0.63
Waist circumference (cm)	76.6 (7.7)	76.6 (7.4)	76.5 (8.0)	0.81	76.9 (7.8)	76.3 (7.7)	0.60
Waist circumference (groups)							
< 88 cm	212 (92.2%)	107 (93.0%)	105 (91.3%)		110 (94.0%)	102 (90.3%)	
≥ 88 cm	18 (7.8%)	8 (7.0%)	10 (8.7%)	0.62	7 (6.0%)	11 (9.7%)	0.29
**Hormonal and reproductive history**							
Hormone therapy use (in the past)							
No	158 (68.7%)	77 (67.0%)	81 (70.4%)		73 (64.6%)	85 (72.7%)	
Yes	72 (31.3%)	38 (33.0%)	34 (29.6%)	0.57	40 (35.4%)	32 (27.4%)	0.19
Contraceptive pill use							
Never	104 (45.2%)	55 (47.8%)	49 (42.6%)		43 (38.0%)	61 (52.1%)	
Ever	126 (54.8%)	60 (52.2%)	66 (57.4%)	0.43	70 (62.0%)	56 (47.9%)	0.03
Number of children							
0	40 (17.4%)	22 (19.1%)	18 (15.7%)		19 (16.8%)	21 (17.9%)	
1	94 (40.9%)	52 (45.2%)	42 (36.5%)		50 (44.2%)	44 (37.6%)	
2 +	96 (41.7%)	41 (35.7%)	55 (47.8%)	0.17	44 (38.9%)	52 (44.4%)	0.58
Breastfeeding							
Never	38 (20.0%)	23 (24.7%)	15 (15.5%)		16 (17.0%)	22 (22.9%)	
Ever	152 (80.0%)	70 (75.3%)	82 (84.5%)	0.11	78 (83.0%)	74 (77.1%)	0.31
Age at first child (years)	27.7 (5.1)	27.6 (4.8)	27.8 (5.5)	0.79	27.5 (5.4)	27.9 (4.8)	0.57
Age at menarche (years)	12.6 (1.4)	12.5 (1.5)	12.7 (1.4)	0.33	12.5 (1.5)	12.7 (1.4)	0.19
Age at menopause (years)	49.9 (4.1)	49.9 (4.0)	49.9 (4.1)	0.98	49.1 (8.0)	49.4 (4.8)	0.72
**Women at work**	132 (57.4%)	68 (59.1%)	64 (55.7%)	0.67	65 (58.0%)	67 (57.8%)	0.95
**Physical activity at work**							
Sedentary	80 (60.6%)	46 (67.7%)	34 (53.1%)		39 (60.0%)	41 (61.2%)	
Standing	34 (25.8%)	13 (19.1%)	21 (32.8%)		17 (26.2%)	17 (25.4%)	
Manual	18 (13.1%)	9 (13.2%)	9 (14.1%)	0.25	9 (13.9%)	9 (13.4%)	0.99
**Total physical activity index**^**d**^							
Inactive	60 (26.1%)	37 (32.2%)	23 (20.0%)		32 (28.3%)	28 (23.9%)	
Moderate inactive	58 (25.2%)	25 (21.7%)	33 (28.7%)		29 (25.7%)	29 (24.8%)	
Moderate active	94 (40.9%)	44 (38.3%)	50 (43.5%)		44 (38.9%)	50 (42.7%)	
Active	18 (7.8%)	9 (7.8%)	9 (7.8%)	0.19	8 (7.1%)	10 (8.6%)	0.85

^a^Arms 1 + 3 *versus* arms 2 + 4.

^b^Arms 2 + 3 *versus* arms1 + 4.

^c^P values were calculated from GLM for continuous variables and from X^2^ test for categorical variables.

^d^The total physical activity index is categorized into four levels based on a cross-tabulation of occupational activity by the combined household and recreational activities in quartiles.

### Outcome measures

Concentrations of pro-inflammatory cytokines were measured in plasma samples collected at baseline and at the end of the intervention. Paired samples were blinded and analysed in the same batch in the Department of Experimental and Clinical Medicine laboratory (University of Florence, Florence, Italy). Samples were tested for IL-1α, IL-1β, IL-6, and TNF-α levels by MILLIPLEX MAP kit (EMD Millipore, Massachusetts, USA) and for CRP level by Affymetrix ProcartaPlex Multiplex Immunoassays (Invitrogen, California, USA), based on Luminex xMAP technology, according to the manufacturer’s instructions. The upper and lower limits of quantification (ULOQ-LLOQ) for each tested analyte were as follows: 10,000–9.4 pg/ml for IL-1α, 10,000–0.8 pg/ml for IL-1β, 10,000–0.9 pg/ml for IL-6, 10,000–0.7 pg/ml for TNF-α and 9–0.02 mg/L for CRP. Cytokines were detected using the fluorescent imager MAGPIX System (Luminex, Texas, USA) and data were acquired and analysed by xPONENT 4.2 software. The concentration of the samples was calculated by plotting the expected concentration of the standards against the mean fluorescence intensity generated by each standard. A four-parameter logistic algorithm was used for the best curve fit.

### Statistical analyses

Plasma cytokines concentrations falling below the LLOQ were replaced with one-half the respective LLOQ for descriptive purposes^34,35^. No subject had values above the ULOQ. Cytokines concentration values were log-transformed in order to normalize the distribution and baseline and follow-up geometric means and standard deviations were estimated.

The factorial design allowed to evaluate the effect of dietary or PA interventions separately.

In order to evaluate the effect of the dietary intervention we compared subjects randomised to arms #1 and #3 *versus* arms #2 and #4. To evaluate the effect of the PA intervention we compared subjects randomised to arms #2 and #3 *versus* arms #1 and #4.

The analysis was performed on an intention-to-treat basis. We applied Tobit regression models for left censored variables^36,37^ to evaluate the effect of the dietary and PA interventions on follow-up log-transformed cytokine levels in comparison with the respective control groups. Analyses were adjusted for baseline log-transformed level of each cytokine and randomisation block variables (age and BMI). We also run the analyses adding to the model the weight change occurred during the intervention. Tobit regression coefficients (β), and relative 95% confidence intervals (95% CI), were then back transformed in the exponential form (exp(β)). An exp(β) value less than 1.0 indicates a lower cytokine level in treatment group compared to control group after the 24-month intervention. We also tested the interaction between the two treatments^38^.

The analyses were performed using Stata 14.0 statistical software (StataCorp LLC, College Station, TX).

## Results

Concentrations of pro-inflammatory cytokines in plasma samples collected at baseline and at the end of the 24-month study period are reported in Table 2 together with the number of subjects with baseline cytokine level under LLOQ. No differences between treatment groups emerged in baseline levels of the cytokines considered. Cytokine levels under LLOQ were equally distributed among treatment groups at baseline (all P values for χ^2^ test > 0.05).

**Table 2:** Analysis according to treatment group compared with control group.

	Dietary intervention^a^			Physical activity intervention^b^			
	Treatment group (N = 115)	Control group (N = 115)	P value^f^	Treatment group (N = 113)	Control group (N = 117)	P value^f^	P interaction between treatments
**TNFα (pg/ml)**							
2 subjects (0.9%) with baseline level under LLOQ^c^							
Baseline geometric mean (95% CI)	7.13 (6.48–7.86)	7.54 (6.66–8.54)	0.15	7.48 (6.78–8.25)	7.20 (6.37–8.14)	0.96	0.70
Follow-up geometric mean (95% CI)	7.52 (6.78–8.33)	8.25 (7.39–9.21)		7.88 (7.13–8.71)	7.87 (7.03–8.80)		
Follow-up adjusted geometric mean (95% CI)^d^	7.52 (7.02–8.05)	8.22 (7.52–8.98)		7.88 (7.34–8.46)	7.83 (7.18–8.55)		
Exp(β) (95% CI)^e^	0.95 (0.86–1.05)	1.00		0.98 (0.88–1.09)	1.00		
P value	0.34			0.69			
**IL-1α (pg/ml)**							
82 subjects (35.7%) with baseline level under LLOQ^c^							
Baseline geometric mean (95% CI)	30.84 (22.28–42.70)	42.61 (31.33–57.94)	0.90	32.76 (23.71–45.26)	39.98 (29.27–54.61)	0.72	0.88
Follow-up geometric mean (95% CI)	56.01 (41.57–75.45)	54.84 (41.66–72.21)		44.57 (33.92–58.56)	68.41 (51.00–91.77)		
Follow-up adjusted geometric mean (95% CI)^d^	36.70 (27.37–49.21)	38.11 (29.06–49.97)		27.66 (20.91–36.61)	50.04 (38.04–65.81)		
Exp(β) (95% CI)^e^	1.27 (0.85–1.90)	1.00		0.66 (0.44–0.98)	1.00		
P value	0.24			0.04			
**IL-1β (pg/ml)**							
68 subjects (29.6%) with baseline level under LLOQ^c^							
Baseline geometric mean (95% CI)	1.76 (1.42–2.18)	1.73 (1.40–2.12)	0.38	1.58 (1.27–1.96)	1.92 (1.56–2.35)	0.84	0.24
Follow-up geometric mean (95% CI)	2.18 (1.84–2.58)	2.26 (1.92–2.67)		1.94 (1.67–2.26)	2.52 (2.11–3.02)		
Follow-up adjusted geometric mean (95% CI)^d^	1.72 (1.45–2.05)	1.81 (1.54–2.13)		1.50 (1.27–1.77)	2.07 (1.76–2.43)		
Exp(β) (95% CI)^e^	0.93 (0.74–1.18)	1.00		0.84 (0.66–1.06)	1.00		
P value	0.58			0.14			
**IL-6 (pg/ml)**							
68 subjects (29.6%) with baseline level under LLOQ^c^							
Baseline geometric mean (95% CI)	2.00 (1.58–2.53)	2.07 (1.64–2.60)	0.52	1.97 (1.55–2.50)	2.10 (1.67–2.63)	0.93	0.34
Follow-up geometric mean (95% CI)	2.97 (2.40–3.68)	2.90 (2.37–3.55)		2.48 (2.03–3.03)	3.44 (2.79–4.26)		
Follow-up adjusted geometric mean (95% CI)^d^	2.42 (2.00–2.92)	2.34 (1.96–2.79)		1.95 (1.62–2.34)	2.89 (2.43–3.45)		
Exp(β) (95%CI)^e^	1.05 (0.80–1.39)	1.00		0.70 (0.53–0.93)	1.00		
P value	0.70			0.01			
**CRP (mg/l)**							
32 subjects (13.9%) with baseline level under LLOQ^c^							
Baseline geometric mean (95% CI)	0.22 (0.16–0.29)	0.20 (0.15–0.27)	0.82	0.19 (0.15–0.26)	0.23 (0.17–0.30)	0.62	0.78
Follow-up geometric mean (95% CI)	0.25 (0.20–0.33)	0.27 (0.20–0.35)		0.25 (0.19–0.32)	0.27 (0.21–0.36)		
Follow-up adjusted geometric mean (95% CI)^d^	0.23 (0.18–0.30)	0.24 (0.18–0.31)		0.23 (0.18–0.29)	0.25 (0.19–0.32)		
Exp(β) (95% CI)^e^	0.92 (0.73–1.15)	1.00		1.04 (0.83–1.31)	1.00		
P value	0.45			0.72			

The DAMA (Diet, Exercise and Mammography) trial.

^a^Arms #1 + #3 *versus* arms #2 + #4.

^b^Arms #2 + #3 *versus* arms #1 + #4.

^c^LLOQ = lower limit of quantification. Plasma cytokines concentrations falling below the LLOQ were replaced with LLOQ/2.

^d^Adjusted for log-transformed cytokine level at baseline and randomisation block variables (age and BMI).

^e^Tobit model adjusted for baseline log-transformed cytokine level and randomisation block variables (age and BMI).

^f^P values according to treatments were calculated from GLM.

Overall, we observed a tendency to an increase in the concentration of each cytokine at the end of the study period. However, when we compared the follow-up values of each cytokine by treatment groups in adjusted models, women randomised to PA intervention (arms #2 + #3) showed lower levels of each cytokine in comparison with women not randomised to this intervention (arms #1 + #4), with exp(β) below 1.00, except for CRP and TNF-α for which we observed exp(β) values around 1. In particular the effect of PA intervention emerged for IL-1α (exp(β) 0.66; 95% CI 0.44–0.98; P = 0.04) and IL-6 (exp(β) 0.70; 95% CI 0.53–0.93; P = 0.01). For IL-1β the effect was less evident (exp(β) 0.84; 95% CI 0.66–1.06; P = 0.14).

No differences in cytokine levels at follow-up emerged in women randomised to the dietary intervention (arms #1 + #3) in comparison with women not randomised to that intervention (arms #2 + #4). All exp(β) were close to 1 (Table 2).

Overall, participants experienced a 0.34 kg weight loss (SD 2.9). The weight loss was more evident in the dietary intervention arm (0.48 kg; SD 3.3) and less evident in the control arm (0.16 kg; SD 2.5). When the analyses were further adjusted for weight change occurred during the intervention, results were materially unchanged (data not shown).

No interaction effect emerged between dietary and PA treatments for any cytokine (Table 2).

### Compliance with the proposed interventions

The baseline and end of study reported dietary and PA habits are shown in Table 3 by treatment. No differences were evident at baseline except for some differences in reported consumption of cheese and cakes and in estimated energy intake by PA treatment. A higher vegetables, legumes and extra virgin olive oil consumption and a lower red meat consumption emerged at the end of the study among participants randomised to the dietary intervention group in comparison with the control group. We also found an increase in reported MET-hours/week of recreational activities in the PA intervention group in comparison with the control group (Table 3).

**Table 3:** Consumption of selected food, alcohol intake, kilocalories and leisure time physical activity level at baseline and at the end of the intervention by treatments.

		Dietary intervention^a^			Physical activity intervention^b^		
		Treatment Group (N = 115)	Control Group (N = 115)	P value^c^	Treatment Group (N = 113)	Control Group (N = 117)	P value^c^
**Diet**							
Total vegetables (g/day)	Baseline	199.6 (77.1)	199.7 (109.5)	0.99	203.6 (93.9)	195.9 (95.3)	0.54
	Follow-up	241.7 (110.6)	195.8 (100.2)	0.001	218.9 (116.6)	218.6 (99.0)	0.98
Fruit (g/day)	Baseline	321.9 (160.2)	345.2 (182.6)	0.30	330.9 (153.6)	336.1 (188.4)	0.82
	Follow-up	319.7 (141.3)	349.4 (175.7)	0.16	331.9 (154.1)	337.2 (165.7)	0.80
Nuts, seeds and dried fruit (g/day)	Baseline	2.5 (4.7)	2.6 (3.6)	0.95	2.8 (4.6)	2.3 (3.7)	0.40
	Follow-up	4.9 (6.4)	3.4 (5.2)	0.047	3.7 (5.3)	4.5 (6.4)	0.27
Legumes (g/day)	Baseline	20.8 (17.0)	17.7 (11.8)	0.10	18.1 (13.4)	20.4 (15.9)	0.25
	Follow-up	37.9 (25.8)	20.7 (15.3)	< 0.0001	27.3 (21.5)	31.3 (23.9)	0.18
Red and processed meat (g/day)	Baseline	76.7 (40.4)	78.6 (47.7)	0.74	49.8 (47.9)	75.5 (40.2)	0.46
	Follow-up	27.9 (21.5)	51.4 (34.9)	< 0.0001	40.4 (30.2)	38.9 (32.3)	0.72
Cheese (g/day)	Baseline	48.1 (35.1)	43.1 (32.4)	0.27	50.1 (35.5)	41.2 (31.7)	0.05
	Follow-up	30.7 (25.3)	34.8 (21.3)	0.19	35.2 (26.6)	30.4 (19.7)	0.12
Cakes and cookies (g/day)	Baseline	44.7 (60.2)	39.4 (37.8)	0.42	50.8 (59.0)	33.6 (38.4)	0.01
	Follow-up	30.2 (47.1)	38.0 (62.7)	0.29	35.1 (43.2)	33.1 (65.3)	0.78
Extra virgin olive oil (g/day)	Baseline	27.5 (10.9)	28.2 (14.5)	3.69	28.3 (12.7)	27.4 (12.9)	0.62
	Follow-up	31.8 (13.8)	27.5 (13.8)	0.02	29.1 (14.4)	30.1 (13.6)	0.60
Wine (g/day)	Baseline	71.8 (98.9)	72.3 (101.3)	0.97	66.3 (94.7)	77.6 (104.8)	0.39
	Follow-up	55.4 (74.3)	70.1 (96.0)	0.19	55.9 (75.7)	69.4 (94.6)	0.23
Alcohol (g/day)^e^	Baseline	7.8 (10.4)	7.6 (9.8)	0.93	7.2 (9.4)	8.8 (10.8)	0.44
	Follow-up	6.0 (8.2)	7.7 (9.4)	0.15	6.0 (7.6)	7.6 (9.8)	0.16
Energy intake (Kcal/day)	Baseline	2082.2 (652.1)	2078.7 (606.3)	0.97	2165.2 (697.5)	1998.6 (543.7)	0.04
	Follow-up	1851.1 (566.7)	2028.6 (698.0)	0.03	1961.2 (659.8)	1919.6 (623.6)	0.62
**Physical activity levels**							
Leisure time activity^d^ (MET-hours/week)	Baseline	88.8 (45.4)	92.6 (48.8)	0.54	88.9 (49.7)	92.4 (44.6)	0.57
	Follow-up	98.6 (47.8)	96.9 (46.5)	0.79	103.5 (44.9)	92.2 (48.6)	0.07
- Household activity (MET- hours/week)	Baseline	63.1 (40.3)	65.2 (41.2)	0.71	63.3 (43.5)	65.0 (37.9)	0.76
	Follow-up	65.8 (40.0)	62.8 (37.0)	0.55	65.0 (36.6)	63.7 (40.3)	0.80
- Recreational activity (MET-hours-week)	Baseline	25.6 (17.7)	24.4 (22.8)	0.51	25.6 (19.7)	27.4 (21.0)	0.49
	Follow-up	32.7 (24.4)	34.1 (23.3)	0.67	38.5 (22.9)	28.4 (23.9)	0.001

Mean values and standard deviations are reported. The DAMA (Diet, Exercise and Mammography) trial.

^a^Arms #1 + #3 *versus* arms #2 + #4.

^b^Arms #2 + #3 *versus* arms #1 + #4.

^c^P values were calculated from GLM comparing each intervention group with the its control group.

^d^Including household and recreational activities.

^e^Alcohol intake from all alcoholic beverages.

## Discussion

In this secondary analysis of the DAMA 24-month trial in healthy postmenopausal women, we observed that at the end of the intervention, women randomised to the PA treatment showed lower plasma levels of pro-inflammatory cytokines IL-1α and IL-6 in comparison with control women, while no significant effect emerged for TNF-α, IL-1β and CRP levels. No effect of the isocaloric dietary intervention on cytokine levels emerged. All these results were confirmed after further adjustment for weight change occurred during intervention. No evidence of interaction emerged between the dietary and the PA intervention thus supporting the independent effect of the PA on the cytokine plasma levels.

Data collected through questionnaires completed at baseline and at the end of the study period showed a change in dietary and physical activity habits in agreement with the objective of the treatment assigned. In particular, women randomised to physical activity treatment showed at the end of the study a significant increase in recreational activities in comparison with the control group. An increase in the consumption of specific foods, namely total vegetables, nuts and seeds, legumes and extra-virgin olive oil and a decrease in the consumption of red meat emerged in women randomised to the dietary intervention.

To our knowledge most of the studies investigating the association between diet and/or physical exercise and circulating levels of cytokines in postmenopausal women were focused on women with metabolic syndrome or overweight, two conditions that are associated with baseline higher level of CRP and other inflammatory markers^39–41^.

Some randomised trials^23,24,26,42^ were focused on overweight or obese postmenopausal women. The ALPHA trial^24^ and the trial by Campell et al.^26^ reported a reduction in CRP levels in women randomised to a 1-year long PA intervention while no effect emerged for IL-6 and other cytokines examined. The CRP reduction appeared dependent on fat loss and proportional to the exercise volume. In a trial in which a six-month intervention focused on sedentary, overweight/obese women with elevated blood pressure was applied, no effects on CRP plasma level emerged in women randomised to one of three exercise groups characterized by increasing energy expenditure while a reduction in CRP emerged in women who experienced a weight loss > 2.6 kg irrespective of the intervention group assignment^42^. In the NEW trial a reduction in plasma level of CRP and IL-6 was observed in women randomised to a caloric restriction diet with or without exercise, while no effect on biomarkers emerged in women randomised to exercise^23^.

Other trials were focused on postmenopausal women with metabolic syndrome. Kemmler et al. evaluated the effect of a 12-months exercise intervention including both high-intensity strength and endurance exercises and no differences in CRP levels emerged between women randomised to exercise and the control group in which a very low intensity weekly exercise was proposed^43^. In a randomised factorial trial, in which the effects of PA and of a dietary intervention aimed to reduce total and saturated fats and cholesterol intake on the changes in CRP levels were evaluated, the authors reported a reduction of inflammatory biomarkers only in women randomised to the dietary intervention or dietary + PA intervention arms. This intervention was not specifically aimed to reduce the weight of participants and analyses were adjusted for weight change^21^. Another study carried out in subjects with metabolic syndrome showed, after two years, a reduction in serum concentration of CRP, IL-6, IL-7 and IL-8 together with a reduction in metabolic syndrome components including body weight, in the intervention arm in which a Mediterranean style diet was proposed in comparison with the control arm in which only general advice was offered^44^.

Differences in the targeted population, duration of the intervention, and protocols adopted make difficult to compare studies, however, in our trial a lower level of IL-6 at FU emerged in the PA treatment group compared to the control group in agreement with other studies. Interestingly, mean IL-6 level among our participants is consistent with expected plasma concentration among healthy subjects. The plasma IL-6 level in resting healthy subjects was about 1 pg/ml, while a chronic low level increase (< 10 pg/ml) was associated with several pathologic conditions and with low PA levels. Moreover, a negative association was documented between IL-6 plasma levels and the amount of regular PA level. IL-6 seems indeed to be down-regulated by PA training^45^. On the other hand we did not find a reduction in CRP plasma values. Again, in our study on healthy women, CRP plasma levels were below the value of 3 mg/L usually considered the threshold level for “normal” values^10^ and we did not register a weight loss as reported in other trials.

All participants in the trial were characterized by a high BD, a well known breast cancer risk factor. However scanty data are available on the possible association between inflammatory cytokines and high BD. In a study where the expression of inflammatory markers in normal breast tissue of breast cancer patients was evaluated in immunohistochemical stained slides, women having higher expression of pro-inflammatory cytokines had higher BD while anti-inflammatory markers were associated with lower BD^46^. However, in a large cross-sectional study in postmenopausal women no association emerged between plasma levels of IL-6, TNF-α and CRP and BD^47^. An inverse association emerged in a recent study carried out in Chinese women^48^.

Strengths of the DAMA trial include an approach aimed to obtain a general modification of dietary and PA habits in the daily life for a relatively long period through practical activities, group meetings and individual counselling. We monitored the compliance of participants, although the use of self reported dietary and PA information represents a limit of this study in comparison to more objective measures.

Participants in the DAMA trial were healthy women not affected by metabolic syndrome and mainly non overweight or obese thus with low inflammatory markers levels at baseline. These characteristics represent at the same time a strength and a limitation of the study. The strength is represented by the possibility to evaluate the effect of general modifications of dietary and PA habits among healthy individuals, while in previous studies participants were mainly obese or affected by metabolic syndrome. The limitation is represented by the general low inflammatory markers level that make it difficult to detect further decreases. Actually, we observed a slowdown in the inflammatory markers level increase in women randomised to the PA treatment.

We observed a lack of effect of our Mediterranean style isocaloric dietary intervention on all the examined cytokines. We carried out a dietary intervention aimed to a comprehensive modification of diet, providing indication on food choices and frequency of consumption. No indications were provided concerning the amount of foods to be consumed with the aim to avoid a weight change, being aware of the effect of body fat on BD measurements, the main outcome of the study^29^. The lack of results of our dietary intervention is probably due to this isocaloric protocol while, in Mediterranean countries dietary modifications probably must lead to some weight loss to achieve results in terms of biomarkers changes.

Overall we observed a tendency to an increase of each cytokine in study subjects over the 24-months of the study irrespective of the treatment assigned. This suggests that even in healthy people with low baseline cytokine levels, a detectable although modest increase in inflammatory markers might occur in a relatively short time period, thus supporting the hypothesis of the age related modification of the cytokine expression pattern that has been reported as an underlying molecular mechanism for a series of chronic diseases including cancer^49,50^. Lifestyle habits such as PA and diet and their changes across the life span may play a role in modifying this process.

Chronic low-grade inflammatory state, is a condition in which inflammatory processes are chronically stimulated at low levels affecting the entire organism. The link between chronic low-grade inflammation and various chronic diseases is now evident and the control of this condition is probably important for the prevention of the most common diseases in the general population^1,2^.

In conclusion this study support the effectiveness of PA, even at moderate intensity, in reducing the increase of inflammatory markers over time and the possibility to reduce the risk of chronic diseases in postmenopausal women. Primary prevention activities aimed to the modification of lifestyle habits are becoming increasingly important in reducing chronic low-grade inflammatory processes.

## Data Availability

The datasets generated during and/or analysed during the current study are not publicly available due to participants privacy protection but are available from the corresponding author on reasonable request.
